# Novel Disperse Dyes Based on Enaminones: Synthesis, Dyeing Performance on Polyester Fabrics, and Potential Biological Activities

**DOI:** 10.3390/molecules29102227

**Published:** 2024-05-09

**Authors:** Khaled M. A. Abdelmoteleb, Ashraf A. F. Wasfy, Morsy Ahmed El-Apasery

**Affiliations:** 1 Chemistry Department, Faculty of Science, Benha University, Banha 13511, Egypt; 2 Dyeing, Printing and Textile Auxiliaries Department, Textile Research and Technology Institute, National Research Centre, 33 El Buhouth St., Dokki, Cairo 12622, Egypt

**Keywords:** azo disperse dyes, fastness properties, dye bath reuse, biological activities

## Abstract

1-(3-aryl)-3-(dimethylamino)prop-2-en-1-one (enaminones) derivatives and the diazonium salt of *para*-chloroaniline were used to synthesize several novel disperse azo dyes with high yield and the use of an environmentally friendly approach. At 100 and 130 °C, we dyed polyester fabrics using the new synthesized disperse dyes. At various temperatures, the dyed fabrics’ color intensity was assessed. The results we obtained showed that dyeing utilizing a high temperature method at 130 °C was enhanced than dyeing utilizing a low temperature method at 100 °C. Reusing dye baths once or twice was a way to achieve two goals at the same time. The first was obtaining a dyed product at no cost, and the second was a way to treat the wastewater of dyeing bath effluents and reuse it again. Good results were obtained for the fastness characteristics of polyester dyed with disperse dyes. When the disperse dyes were tested against certain types of microbes and cancer cells, they demonstrated good and encouraging findings for the potential to be used as antioxidants and antimicrobial agents.

## 1. Introduction

**Figure 1:**
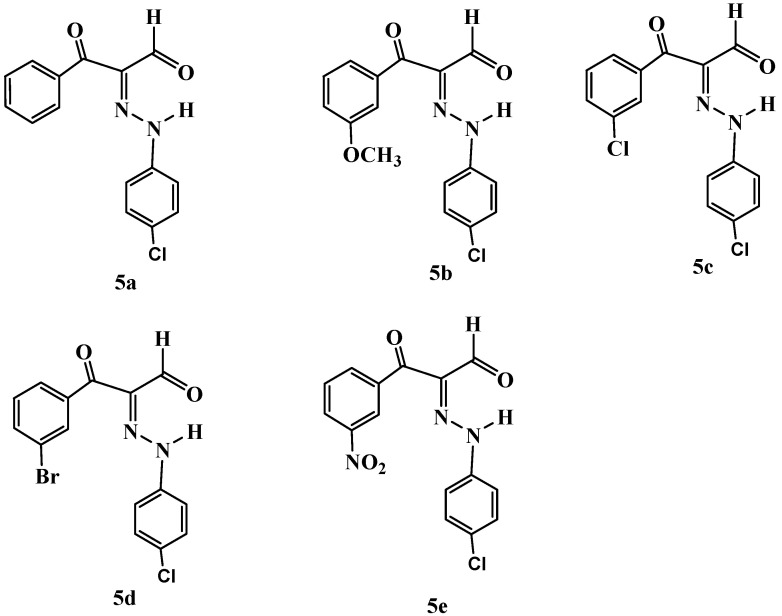
Chemical structure of the synthesized dyes.

Since ancient times, natural dyes have played an important role in the lifestyle and beliefs of people and have gained their appreciation and respect. The dye industry has grown rapidly in general but especially in the last twenty years after the great demand for it has increased [1,2,3]. Organic substances called azo dyes account for more than half of the dyes now used. Since azo dyes have a variety of useful qualities, they are frequently used in industrial dyes [4,5,6]. Azo dyes are essential. They have many uses and are widely used in a variety of industries, including the food, medical, pharmaceutical, leather, and textile industries [7]. These dyes are also utilized in various processes, such as photosensitive dyes and thermal and laser printing. Polyester is a hydrophobic fiber. This leads to the use of disperse dyes in its aqueous dyeing, which can be carried out at high or moderate temperatures [8,9,10,11]. Where does the newness of life come from? The answer to this question is manufacturing processes because this is the main tool for the economic growth of nations because it plays a major role in the economic development of any country. The textile industry sector is of utmost importance, not only because it has many diverse job opportunities but because this industry is also able to create integration with other economic sectors. Therefore, the textile industry sector is considered the most expanded and widespread sector. Among the main raw materials on which the textile industry sector depends are synthetic dyes, especially azo dyes, synthetic fibers, especially polyester fibers, and treated water. Therefore, the synthesis of new azo dyes and the possibility of treating effluents is the solid foundation of this industry [12,13,14,15]. If we shed light on the liquid waste coming out of the textile industry, it comprises a large amount of color left over from dyeing and printing processes, suspended solids, dissolved solids, and salt. The effluent is more polluted due to the presence of dyes and chemicals in the printing, dyeing, and printing processes, which are not easily degraded using conventional processing methods. To remove pollutants from liquid waste in textiles, work has been carried out to develop different treatment techniques, such as chemical, biological, and physical treatment, or combining them; this requires a lot of money, effort, and time [16,17,18,19]. When auxiliary chemicals are used for more than one dyeing cycle or two dyeing cycles, in this case, the dye bath can be used again for dyeing once or more [8,20,21,22,23,24,25,26,27,28,29,30,31,32,33,34,35,36,37,38]. This lowers the cost of dyeing, which in turn reduces the amount of wastewater and pollutants discharged. It is also a simple approach to the treatment of water used after the dyeing process. To our knowledge, no research has been conducted on the use of enaminone derivatives in the creation of disperse dyes, as can be seen from the literature survey [1,25,30,31], except for what we have published since 2015, but in 2017, Alassar et al. [39] created a variety of disperse dyes for dyeing polyester using enaminone derivatives. Since 2015, we have focused on creating an environmentally friendly and innovative program for synthesizing various disperse dyes based on enaminones, known for their exceptional brightness in deeper and darker shades. They also have a greater brightness than their aromatic or benzene counterparts. In this study, novel disperse dyes based on enaminone derivatives were synthesized via coupling the diazonium salt of *para*-chloroaniline with synthesized enaminones (Figure 1). These disperse dyes were utilized to dye substrate (polyester fabrics) via low temperature method at 100 °C or via high temperature method at 130 °C. We accomplished this by assessing dyeing efficiency using K/S values to determine the color intensity of the dyed polyester materials. These dyes were used to assess the polyester fabrics’ fastness characteristics.

The innovation of this study lies in the production of a number of new disperse dyes based on enaminones, which contain substituted groups that give or withdraw electrons, which give them different bright colors; then, we used these new dyes in dyeing polyester fabrics and finally investigated the possibility of these new synthetic dyes having activity against some bacteria, fungi, and cancers; this gives them added value.

## 2. Results and Discussions

### 2.1. Chemistry

**Scheme 1:**
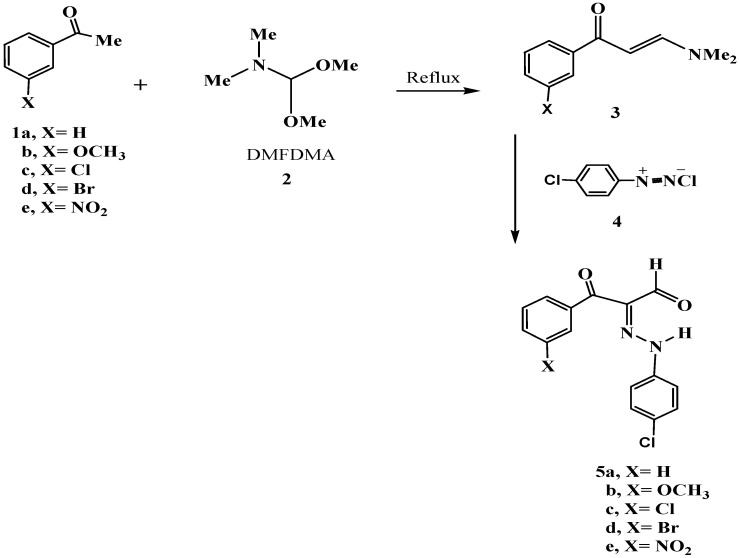
Synthesis of dyes **5a**–**e**.

**Figure 2:**
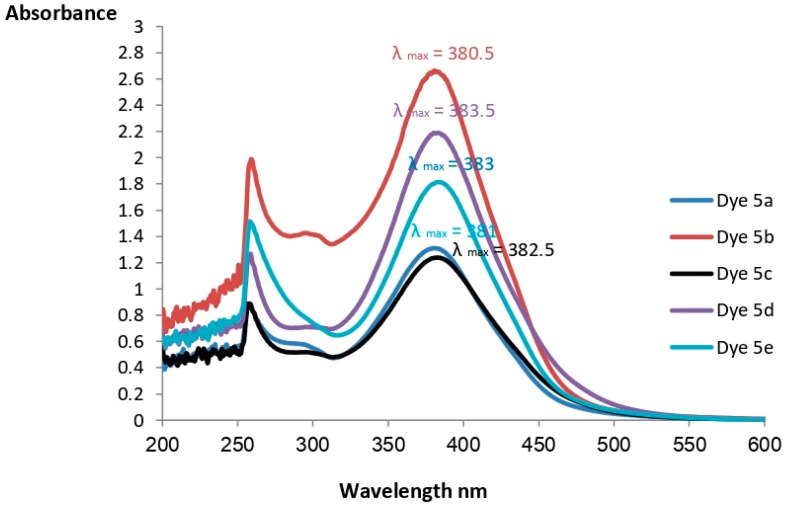
Absorption spectrum in DMSO for the dyes.

We can define any substance that contains an enamine group in addition to a carbonyl group by the term 1-(3-aryl)-3-(dimethylamino)prop-2-en-1-ones (enaminones). It is worth noting that enaminones serve as excellent building blocks for larger organic molecules because they contain many functional groups. Enaminones can be used to create a wide range of organic molecules for industrial purposes such as the production of many different dyes. Despite the very large number of structures made from enaminones in many different chemical industries, enaminones themselves are not easily synthesized; a catalyst is often required. In order to overcome the activation energy, many chemical reactions require an organic solvent, which is often harmful, but the synthesis of the enaminones that we successfully prepared did not require solvents. The multiple functional groups in enaminones can interact through many different routes. Many compounds can pass through amine chemistry. Weak nucleophiles can undergo 1, 4-addition, the carbon–carbon double bond can be reduced by a wide range of different compounds, and strong nucleophiles can be added to a ketone. Complementing our strategy toward synthesizing new dyes, we synthesized new, biologically active disperse dyes without using any solvents as an environmentally safe approach. In this study, we present the synthesis of these new dyes based on enaminones **3a**–**3e**.Compounds **3a**–**3e** were successfully synthesized by condensing acetophenone, *m*-methoxyacetophenone, *m*-chloroacetophenone, *m*-bromoacetophenone, and *m*-nitroacetophenone **1a**–**e** with Dimethylformamide dimethylacetal (DMFDMA) **2**. Compounds **3a**–**3e** were coupled with the diazonium salt of *p*-chloroaniline **4** to synthesize novel disperse dyes **5a**–**5e** (Scheme 1, Table 1 and Figure 2).

Based on proton ^1^H NMR spectral data, the NH groups for dyes **5a**–**5e** appear at δ 14.55, 14.67, 14.71, 14.70, and 14.83, respectively. CHO groups for dyes **5a**–**5e** appear at δ 10.18, 10.16, 10.18, 10.17, and 10.23, respectively. Finally, aromatic protons for dyes **5a**–**5e** appear at ~δ 7.12 to ~δ 8.96.

### 2.2. Dye Uptake

**Figure 3:**
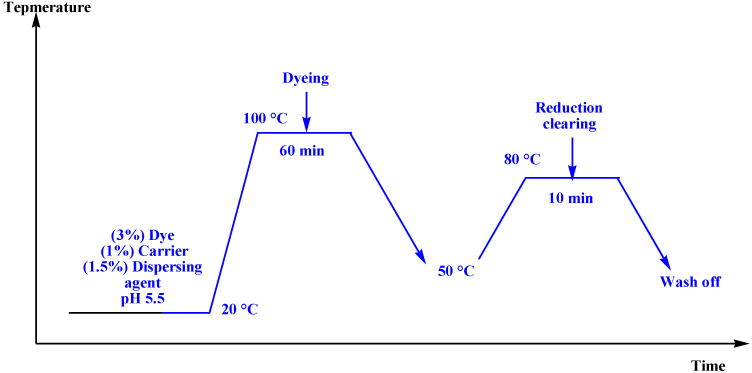
Dyeing graph at 100 °C.

The major type of dye used in coloring polyester fabrics is disperse dyes, which are known to both the dye sciences and textile industries. By using a high temperature of about 130 °C or dyeing at 100 °C with a carrier present, the dyeing rate can be increased to a commercially excellent and consequently extremely acceptable standard. In order to increase dye absorption, speed up dyeing, and reduce dyeing temperature, dyeing polyester using a carrier has been extensively studied (Figure 3).

**Figure 4:**
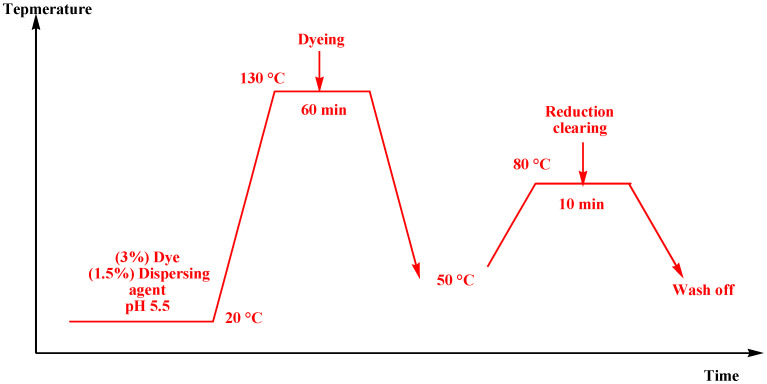
Dyeing graph at 130 °C.

By making the polyester structure more swellable and less compressible, which makes it simpler for the chains to migrate partially and show improved dye absorption, dyeing polyester fabrics at a temperature of 130 °C causes a large rise in K/S values (Figure 4 and Table 2).

The dissolution of the dye molecules and their solubility are further aided by the high temperature, which accelerates and increases the dye’s diffusion and penetrating ability.

According to Table 2’s color strength (K/S) results for the high-temperature dyeing polyesters, disperse dye **5a** has a strong affinity for polyester fabrics and a K/S of 15.51. When the phenyl moiety of compound **5a** was swapped out for an electron-withdrawing group like (C_6_H_4_Cl-*m*, C_6_H_4_Br-*m*, or C_6_H_4_NO_2_-*m*), the resulting compounds **5c**, **5d**, and **5e** had lesser color strengths (K/S = 11.97, 14.31, and 12.14, respectively).

Similar results for the low-temperature dyeing polyesters are shown in Table 3, where the (K/S) values demonstrate that disperse dye **5a** has an excellent affinity for polyester fabrics and a K/S of 14.62. When the phenyl moiety of compound **5a** was swapped out for an electron-withdrawing group like (C_6_H_4_Cl-*m*, C_6_H_4_Br-*m*, or C_6_H_4_NO_2_-*m*), the resulting compounds **5c**, **5d**, and **5e** had lesser color strengths (K/S = 12.75, 11.98, and 11.38, respectively).

The results in Table 2 and Table 3 clearly indicate that the color intensity K/S values of polyester fabrics dyed with the new disperse dyes at a temperature of 130 °C are better than the color intensity K/S values of the polyester fabrics dyed with the new disperse dyes at a temperature of 100 °C. One of the major water-intensive industries is the textile sector, particularly when it comes to wet processes.

### 2.3. Fastness Properties

Polyester fabrics were colored using the synthesized disperse dyes, **5a**–**e**. At 130 °C (Table 4) or 100 °C (Table 5), the dyed polyester textiles performed well for rubbing, reasonably well for light fastness, and extremely well for washing and perspiration fastness. We found that the values of light fastness for polyester fabrics dyed with dyes **5a** to **5e** at 130 °C range from 3–4 to 4, and that the values of light fastness for polyester fabrics dyed with dyes **5a** to **5e** at 100 °C range from 4 to 4–5, which shows that the stability against the light of polyester fabric samples dyed at 100 °C is better than those dyed at 130 °C.

We found that the stability values of rubbing fastness for polyester fabrics dyed at 100 °C were lower than the stability values of rubbing fastness for polyester fabrics dyed at 130 °C, especially for dyes **5b** to **5d**.

We found that the values of washing fastness and perspiration fastness for polyester fabrics dyed with dyes **5a** to **5e** at a temperature of 130 °C were the same as the values of washing fastness and perspiration fastness for polyester fabrics dyed with dyes **5a** to **5e** at a temperature of 100 °C.

### 2.4. Dyebath Reuse

Numerous environmental contaminants are also produced by this sector. We employed a straightforward technique to purify the water used after the dyeing process by recycling the dyeing bath once or twice more for dyeing, which was an inventive step to eliminate contaminants from liquid waste in textiles. As a result, dyeing was much less expensive, which lowered the quantity of wastewater and other pollutants generated that are released into the environment and endanger the ecosystem.

When polyester fabrics were dyed with the prepared disperse dyes at 130 or 100 °C, it was found that the dye effluents contained a significant amount of dye that was harmful to the environment. Therefore, in order to make the most of the dye that was used while preventing the disposal of any colored waste that had a detrimental influence on the environment, our strategy was to employ the dye effluent waste in dyeing new polyester fabrics. Based on the information in Table 6, we can see that for all disperse dyes, the color strength K/S value of the dye bath reuse procedure varied by ~6% and ~1.5% during the first and second re-dyeing processes at 130 °C from its initial value.

Based on the information in Table 7, we can see that for all disperse dyes, the color strength K/S value of the dye bath reuse procedure varied by~30% and ~5% during the first and second re-dyeing processes at 100 °C from its initial value.

Polyester fabrics were colored using the synthesized disperse dyes, **5a**–**e**. At 130 °C (Table 8) or 100 °C (Table 9), we found that the stability values of rubbing fastness for polyester fabrics dyed at 130 °C were lower than the stability values of rubbing fastness for polyester fabrics dyed at 100 °C, especially for dyes **5b** and **5c**.

We found that the values of perspiration fastness and washing fastness for polyester fabrics dyed with dyes **5a** to **5e** at a temperature of 130 °C were the same as the values of perspiration fastness and washing fastness for polyester fabrics dyed with dyes **5a** to **5e** at a temperature of 100 °C.

### 2.5. Antimicrobial Activity

**Figure 5:**
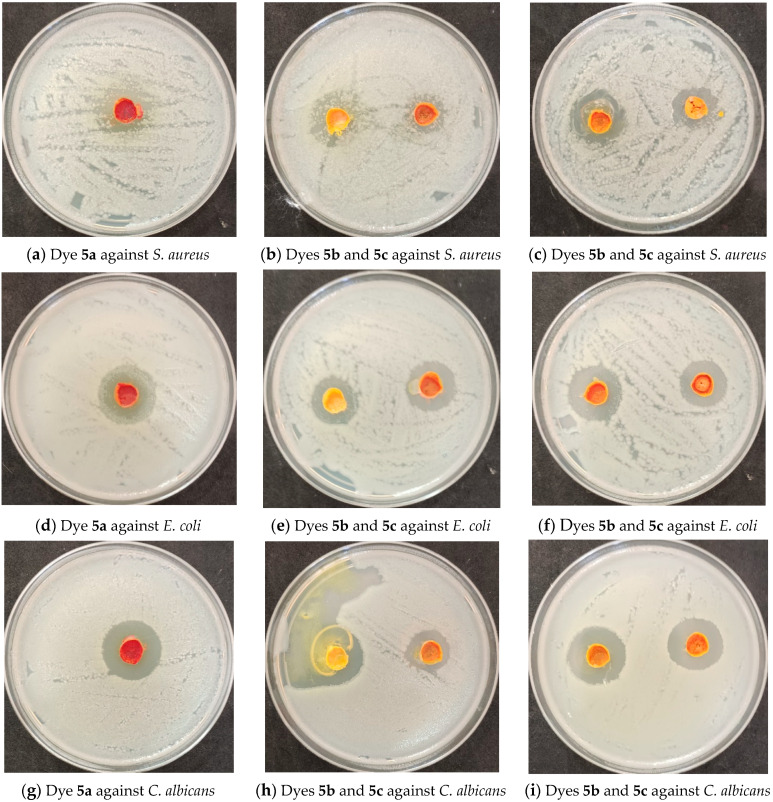
The images display the disperse dyes’ zone of inhibition.

At least three microorganisms are physiologically active against each of the research dyes, as shown in Table 10 and Figure 5. Dyes **5a** and **5d** showed excellent microbial activity against *Candida albicans*, *Escherichia coli*, and *Staphylococcus aureus*, whereas dyes **5b** and **5e** showed very good microbial activity against *Candida albicans*, *Escherichia coli*, and *Staphylococcus aureus*, while dye **5c** showed good microbial activity against *Candida albicans*, *Escherichia coli*, and *Staphylococcus aureus*. On the contrary, dyes **5a** to **5e** did not show any activity against *Aspergillus niger*.

### 2.6. In Vitro Cytotoxicity Evaluation

**Figure 6:**
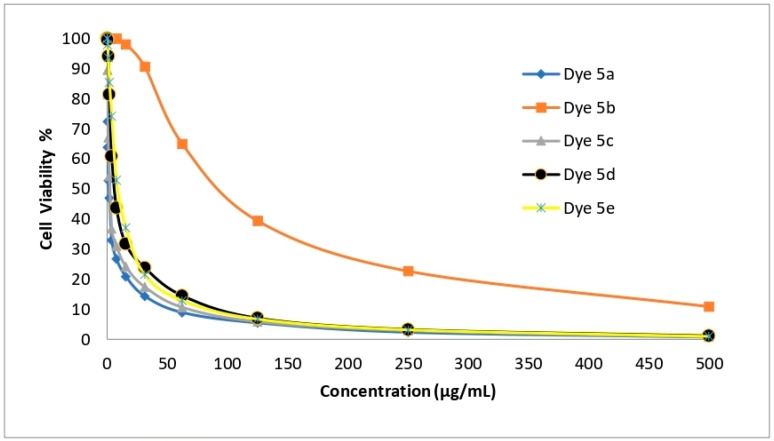
The ability of disperse dyes **5a**–**e** to inhibit the growth of HepG-2 cells.

**Figure 7:**
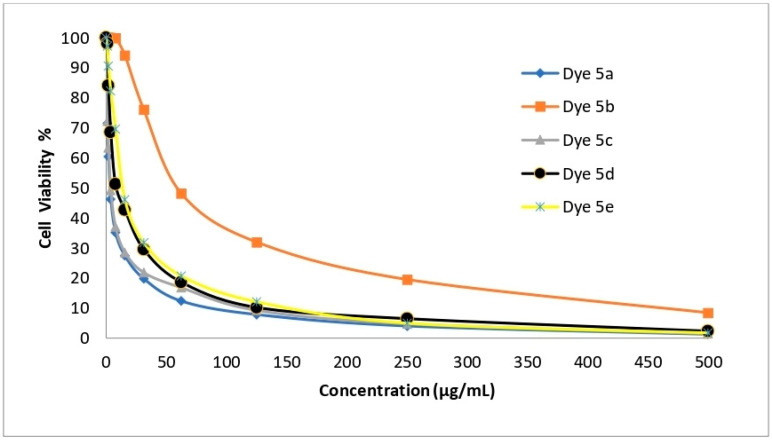
The ability of disperse dyes **5a**–**e** to inhibit the growth of HCT-116 cells.

**Figure 8:**
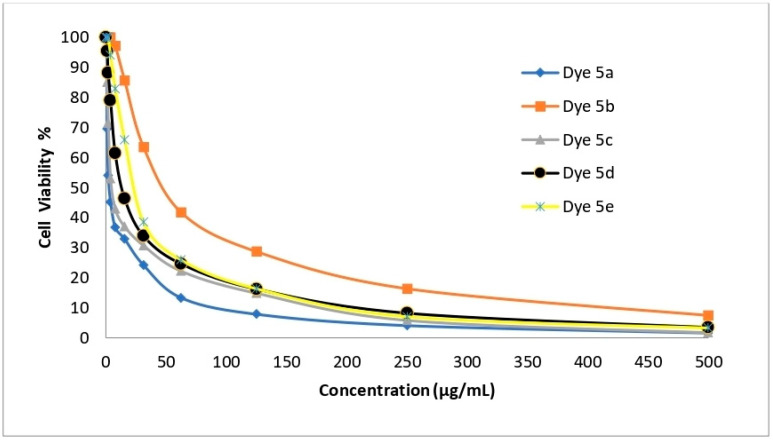
The ability of disperse dyes **5a**–**e** to inhibit the growth of A-549 cells.

**Figure 9:**
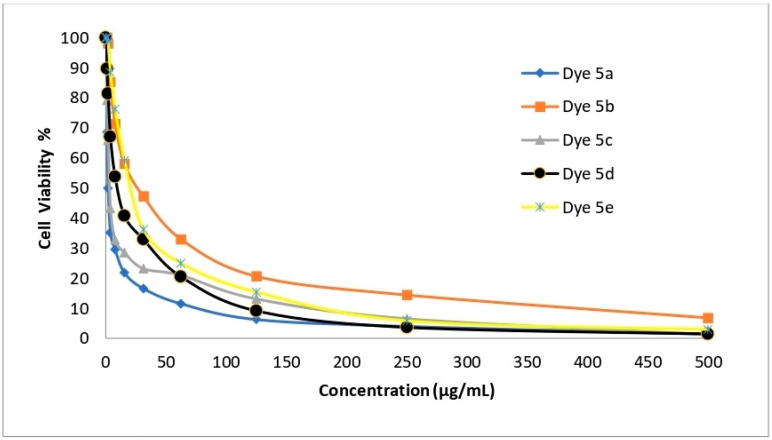
The ability of disperse dyes **5a**–**e** to inhibit the growth of MCF-7 cells.

The newly created disperse dyes **5a**–**e** were tested for their ability to inhibit the growth of four human cell lines: HepG-2 (for hepatocellular carcinoma), HCT-116 (for colon carcinoma), MCF-7 (for breast cancer), and A-549 (for lung cancer). The IC_50_ (g/mL) values—the concentrations needed to prevent 50% of the growth of the culture when the cells are exposed to the tested disperse dyes for 48 h—were calculated using various concentrations of the four disperse dyes. The mean IC_50_ was used to calculate the cytotoxic activity in three different experiments. The data are provided in Table 11 and Figure 6, Figure 7, Figure 8 and Figure 9 which reveal that compounds **5a** and **5c** had the highest cytotoxic activity against the HePG-2, HCT-116, MCF-7, and A-549 cells when compared to cisplatin as a reference drug.

Table 11 and Figure 6, Figure 7, Figure 8 and Figure 9 demonstrate the highly substantial activity of disperse dye **5a**, with IC_50_ values in HePG-2, A-549, HCT-116, and MCF-7 cells of 1.48, 2.91, 3.48, and 1.99 g/mL, respectively. Compounds **5c** and **5d** were created by substituting the phenyl moiety in compound **5a** with electron-withdrawing groups like C_6_H_4_Cl-*m* and C_6_H_4_Br-*m*, and their respective IC_50_ values were 2.48, 5.03, 3.89, and 3.39 and 6.38, 13.67, 8.92, and 10.03 g/mL.

With an IC_50_ similar to cisplatin, this modification successfully generated potent anticancer activity against the four cell lines. Interestingly, compound **5a** had an IC_50_ value of 1.48, 2.91, and 1.99 g/mL in each case vs. 3.69, 7.49, and 5.68 g/mL for cisplatin and was more effective at killing HePG-2, A-549 and MCF-7 cells. Also, compound **5c** had an IC_50_ value of 2.48, 5.03, and 3.39 g/mL in each case vs. 3.69, 7.49, and 5.68 g/mL for cisplatin and was more effective at killing HePG-2, A-549, and MCF-7 cells.

Obstinately, when the phenyl moiety of compound **5a** was substituted by an electron-withdrawing group such as (C_6_H_4_NO_2_-*m*) (IC_50_ = 9.23, 24.60, 14.28, and 21.76) in HePG-2, A-549, HCT-116, and MCF-7 cells, the anticancer activity of dye **5e** was reduced.

### 2.7. In Vitro Cytotoxic Activity toward Normal Human WI-38

**Figure 10:**
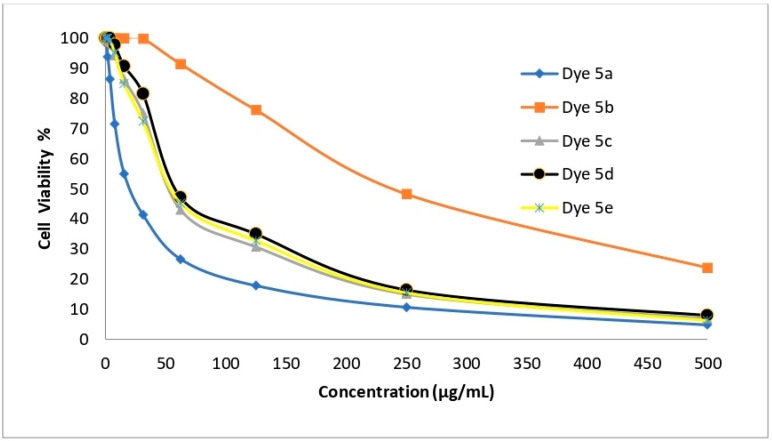
WI-38 human lung fibroblast normal cells.

Disperse dyes **5a**–**5e** were tested for their cytotoxic effect against the normal human lung cell line (WI-38) in order to determine whether the synthesized dyes may be safe for use on normal cells. IC_50_ values were used to express the results (see Table 12 and Figure 10). The cytotoxic impact of disperse dyes **5a**, **5b**, **5c**, **5d**, and **5e** on WI-38 cells was found to have a non-significant cytotoxic impact on (WI-38), with CC_50_ values of 21.23, 241.94, 55.60, 59.82, and 56.91, respectively. Badisa et al. [35] reported that a chemical exhibits specific toxicity to cancer cells when its selective index value is more than two. Conversely, a substance with a selective index value less than two is thought to be generally hazardous, meaning it can also occasion cytotoxicity on normal cells. As anticancer agents, disperse dyes **5a**, **5b**, **5c**, **5d**, and **5e** demonstrated an excellent selectivity index (WI-38/HepG-2, WI-38/A-549, WI-38/HCT-116, and WI-38/MCF-7) range of 2.3 to 22.4, which contributed to their good safety profile.

### 2.8. Antioxidant Activity

**Figure 11:**
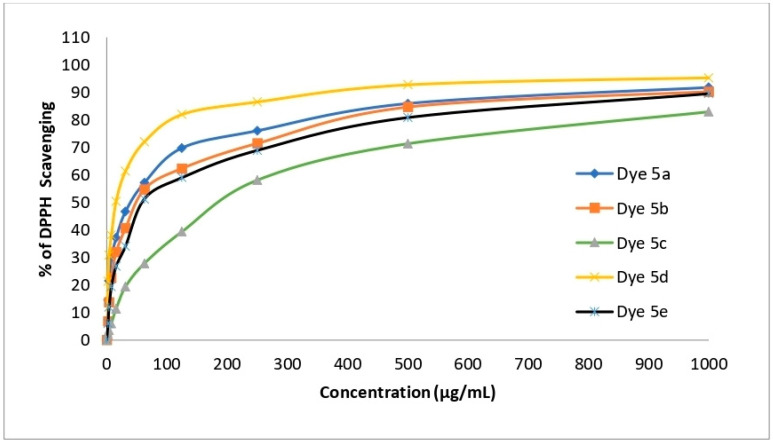
Antioxidant activities of disperse dyes 5a–e.

The ability of the four disperse dyes to scavenge free radicals as well as their antioxidant activity was evaluated in vitro (DPPH). The antioxidant activity of the dyes was measured using the IC_50_ value, which is the dose required to reduce the formation of DPPH radicals by 50%. According to the data in Table 13, disperse dye **5c** has poor antioxidant activity (IC_50_ values of 195.75 when compared to ascorbic acid, which has antioxidant activity (IC_50_ value of 10.62; see Figure 11).

Contrarily, dye **5d** displays extremely potent antioxidant activity, with an IC_50_ of 15.33.

Dye **5a** has very good antioxidant activity, with an IC_50_ of 40.99. Disperse dyes **5b** and **5e** have good antioxidant activity (IC_50_ values of 51.87 and 60.18, respectively).

## 3. Materials and Methods

The NMR spectra were analyzed at 300 MHz using a Mercury-300BB spectrometer (Palo Alto, CA, USA). By comparing material fluctuations to an internal reference of tetramethylsilane (TMS), the ppm findings were recorded at the Faculty of science, Cairo University. The Fourier-transforminfrared (FT-IR) spectra were controlled using a JASCO FT/IR4700 spectrophotometer (Tokyo, Japan). The elemental analyses (C, H, and N) were completed using a PerkinElmer 2400 analyzer (Palo Alto, CA, USA). The solvents used in this exploratory inquiry for both the synthesis methods and the spectroscopic estimations were given by Flukaand Aldrich (Cairo, Egypt).

### 3.1. Preparation of 1-(3-Aryl)-3-(dimethylamino)prop-2-en-1-ones ***3a**–**e***

Following the guidelines provided in our previous work [1], compounds **3a**–**e** were produced. DMFDMA (1.19 g, 0.01 mol) was refluxed for ten hours with acetophenone, *m*-methoxyacetophenone, *m*-chloroacetophenone, *m*-bromoacetophenone, and *m*-nitroacetophenone. Thin-layer chromatography (TLC) was used to assess how successfully the reactions had taken place. Petroleum ether was employed to treat the reaction mixture once it had cooled to room temperature. The solid product that had been produced was filtered and re-crystallized.

### 3.2. Synthesis of Dyes

A cold solution of the diazonium salt 4 [10 mmol] was added to a cold solution of 1-(3-aryl)-3-(dimethylamino)prop-2-en-1-ones **3a**–**e** in ethanol that contained sodium sulfate by adding a cold solution of sodium nitrite (0.69 g) in water to a cold solution of *p*-chloroaniline (10 mmol) in concentrated HCl acid at a temperature of 0 to 5 °C. For one hour, the mixture was stirred at a temperature of 5 °C. The generated solid precipitate was filtered out and crystallized into orange crystals using the proper solvents as listed in Table 1.

### 3.3. Dyeing Procedure

The suitable amount of dye(3% shades) was dissolved in a few drops of dimethylformamide (DMF) to create a dispersion of disperse dyes, which was then added dropwise via stirring to the dye bath (liquor ration: 1:30) containing (1.5%) of dispersing agent and (1%) of an anionic eco-friendly carrier in the case of dyeing at 100 °C or with (1.5%) of dispersing agent in case of dyeing at 130 °C using an infrared dyeing machine (Starlet 3). The polyester textiles were added when the pH of the dye bath was changed to 5.5. At a rate of 3 °C/min, the dye bath had to be heated to 100 °C and held there for 60 min. The dyed polyester materials were first reduced (1 g/L sodium hydroxide and 1 g/L sodium hydrosulfite, 10 min, 80 °C) and then cleaned after being chilled to 50 °C. After rinsing with cold water, the samples were air-dried.

### 3.4. Fastness Properties Tests

According to the American Association of Textile Chemists and Colorists, the blue wool scale (grades 1–8) and grey scale (grades 1–5) were used to evaluate the dyed samples’ fastness properties under various conditions, including rubbing, washing, perspiration, and light fastness [1,25].

#### 3.4.1. Light Fastness

The test is run continuously for 35 h. It may be deduced that the type of fabric into which the dye is put ultimately causes the color of the colored fabric to intensify with increasing dye concentration. This is because different chemical groups present in diverse fabrics can significantly affect a dye’s light resistance index when used on a particular fabric. According to the wavelength dispersion of the light flowing in, not all absorptions start bleaching equally. The rate at which some colorants fade can be significantly influenced by the level of moisture as well as the nature of the environment. The color variations of the examined materials were noted using the blue scale (1–8).

#### 3.4.2. Washing Fastness

To assess washing fastness, the ISO 105-C02 procedure from 1989 was employed [10]. The test pieces were sandwiched between a pair of samples of both wool and cotton fabric, which were then immersed in a solution made up of water with a non-ionic detergent concentration of 5 g/L (1:50) for half an hour at 60 °C. The samples were removed after a predetermined period of time, cleaned twice with sporadic hand pressure, and then dried, with assessments of washing fastness taking place.

### 3.5. Color Strength

The Kubelka–Munk equation was used to calculate the intensity of each color.

$$K/S=\frac{{\left(1-R\;\right)}^{2}}{2R}-\frac{{\left(1-R^{0}\right)}^{2}}{2R^{0}}$$

where K represents the absorption coefficient, S represents the scattering coefficient, R represents the decimal percentage of the dyed cloth’s reflectance, and R^0^ represents the decimal fraction of the non-dyed fabric’s reflectance.

The International Committee on Illumination (CIE) developed the CIELAB (colorspace) psychometric coordinates in 1976. (L*) stands for lightness, while the hue of the dye is expressed as (h*) values and (C*) for chroma. The colorhues of the dyed example were approximated using the CIELAB psychometric coordinates (a*) and (b*), where (a*) denotes the red–green axis and (b*) denotes the yellow–blue axis

### 3.6. Evaluation of the Biological Activities of the Disperse Dyes

The antimicrobial, anticancer, and antioxidant properties of the synthesized dyes were evaluated according to what we recently published [1,9,28].

## 4. Conclusions

This study successfully presents the effective synthesis of novel disperse dyes with high productivity through the reaction of acetophenone derivatives with DMFDMA and then coupling with the diazonium salt of *p*-chloroaniline. These new dyes were used for dyeing polyester fabrics at different temperatures, producing colors ranging from pale orange to orange. A simple, effective method as one of the sustainable treatment methods for water emerging from dyeing baths and the possibility of using it again was investigated. The results showed that dyeing polyester fabrics at high temperatures is environmentally friendly and superior to dyeing at low temperatures, based on the color intensity of the dyed fabrics. The fastness properties of dyed fabrics gave excellent results for their fastness to washing, perspiration, and rubbing and fair results for their light fastness. The outcomes also demonstrated that these new disperse dyes are active against a variety of bacteria and cancer cells. Additionally, the normal cell line (lung WI-38) was used to evaluate the cytotoxic effects of the synthesized dyes, and the dyes showed a satisfactory safety profile as anticancer agents. The outcomes demonstrate the potential of these disperse as antioxidants, antibacterial, and anticancer agents. The above clarification reveals that we have presented comprehensive work not only in preparing a series of new disperse dyes and the possibility of using them in dyeing polyester fabrics but also in demonstrating the added value of these dyes due to their activity against some bacteria and cancers. Also, we present a simple approach that is effective as one of the sustainable wastewater treatment approaches, whereby it can treat the wastewater emerging from the dyeing process and achieve two goals at the same time, the first of which is obtaining no-cost dyed polyester fabrics and the second is the success of the wastewater treatment process and the possibility of using the treated wastewater again in dyeing wet processes, which has a positive impact on the environment if it is thrown into waterways without further treatment processing. To obtain novel effective disperse dyes for future development, compounds **3a** to **3e** require further structural enhancement by reacting dimethylformamide dimethyl acetal with *o*-substituted acetophenone/or ketones. In addition, the synthesis of further derivatives without using solvents in an environmentally safe manner is required.

## Figures and Tables

**Table 1:** Characterization of the new dyes.

Dye No	Name	Yield	I.R. (cm^−1^)	λ_max_ in DMSO *	MS *m*/*z*	^1^H NMR	Elemental Analysis
**5a**	(*E*)-2-(2-(4-chlorophenyl)hydrazono)-3-oxo-3- phenylpropanal	77%	3435, 3160, 1648, 1589	383	(M − 1)^+^ = 385.59	(CDCl_3_): δ 7.24–7.95 (m, 9H, arom-H) 10.18 (s, 1H, CHO), 14.55 (s, 1H, NH).	C_15_H_11_ClN_2_O_2_, Calcd: C, 62.84; H, 3.87; N, 9.77. Found: C, 62.81; H, 3.84; N, 9.84
**5b**	(*E*)-2-(2-(4-chlorophenyl)hydrazono)-3-(3-methoxyphenyl)-3-oxopropanal	80%	3431, 3100, 1640, 1589	380.5	(M − 1)^+^ = 315.44	(CDCl_3_): δ 3.86 (s, 3H, OCH_3_), 7.12–7.53 (m, 8H, arom-H) 10.16 (s, 1H, CHO), 14.67 (s, 1H, NH).	C_16_H_13_ClN_2_O_3_, Calcd: C, 60.67; H, 4.14; N, 8.84. Found: C, 60.41; H, 4.19; N, 8.92.
**5c**	(*E*)-3-(3-chlorophenyl)-2-(2-(4-chlorophenyl)hydrazono)-3-oxopropanal	71%	3431, 2979, 1645, 1568	382.5	(M − 2)^+^ = 319.73	(CDCl_3_): δ 7.27–8.92 (m, 8H, arom-H), 10.18 (s, 1H, CHO), 14.71 (s, 1H, NH).	C_15_H_10_C_l2_N_2_O_2_, Calcd: C, 56.10; H, 3.14; N, 8.72. Found: C, 56.21; H, 3.05; N, 8.66.
**5d**	(*E*)-3-(3-bromophenyl)-2-(2-(4-chlorophenyl)hydrazono)-3-oxopropanal	73%	3432, 2919, 1646, 1522	383.5	(M)^+^ = 365.19	(CDCl_3_): δ 7.26–8.11 (m, 8H, arom-H), 10.17 (s, 1H, CHO), 14.70 (s, 1H, NH).	C_15_H_10_BrClN_2_O_2,_Calcd: C, 49.28; H, 2.76; N, 7.66. Found: C, 49.32; H, 2.73; N, 7.71.
**5e**	(*E*)-2-(2-(4-chlorophenyl)hydrazono)-3-(3-nitrophenyl)-3-oxopropanal	84%	3431, 2918, 1644, 1579	381	(M − 1)^+^ = 330.77	(CDCl_3_): δ 7.27–8.96 (m, 8H, arom-H), 10.23 (s, 1H, CHO), 14.83 (s, 1H, NH).	C_15_H_10_ClN_3_O_4_, Calcd: C, 54.31; H, 3.04; N, 12.67. Found: C, 54.23; H, 3.11; N, 12.62

* 1 mg of dye was completely dissolved in 10 mL of dimethyl sulfoxide (DMSO) (weight/volume); see Figure 2.

**Table 2:** Color strengths at 130 °C.

Dye No.	K/S	λ_max_	L*	C*	a*	h*	b*
**5a**	15.51	380	71.68	68.55	4.89	85.91	68.37
**5b**	14.33	380	72.01	67.51	1.54	88.69	67.49
**5c**	11.97	380	74.01	70.08	0.24	89.80	70.08
**5d**	14.31	380	74.58	63.14	−3.86	93.51	63.02
**5e**	12.14	380	73.00	67.36	1.69	88.56	67.33

**Table 3:** K/S values of the dyed substrate at 100 °C.

Dye No.	K/S	λ_max_	L*	C*	a*	h*	b*
**5a**	14.62	380	65.75	67.78	14.88	77.32	66.13
**5b**	14.00	380	71.82	65.97	2.33	87.98	65.93
**5c**	12.75	380	74.73	65.10	−2.76	92.43	65.04
**5d**	11.98	380	73.96	68.73	0.21	89.83	68.73
**5e**	11.38	380	72.96	63.53	0.00	90.00	63.53

**Table 4:** Fastness properties of the dyed substrate at 130 °C.

Dye No	Light Fastness	Rubbing Fastness		Washing Fastness				Perspiration Fastness							
								Acid				Alkaline			
		Dry	Wet	St.	St. *	St. **	Alt.	St.	St. *	St. **	Alt.	St.	St. *	St. **	Alt.
**5a**	3–4	3–4	3–4	4	4	4	4	4–5	4–5	4–5	4	4–5	4–5	4–5	4
**5b**	4	4	4	4	4	4	4	4–5	4–5	4–5	4	4–5	4–5	4–5	4
**5c**	4	4	3–4	4	4	4	4	4–5	4–5	4–5	4	4–5	4–5	4–5	4
**5d**	3–4	4	4	4	4	4	4	4–5	4–5	4–5	4	4–5	4–5	4–5	4
**5e**	3–4	3–4	3–4	4	4	4	4	4–5	4–5	4–5	4	4–5	4–5	4–5	4

St.: staining on cotton; St. *: staining on wool; St. **: staining on polyester, Alt.: alteration.

**Table 5:** Fastness properties of the polyester fabrics dyed at 100 °C.

Dye No	Light Fastness	Rubbing Fastness		Washing Fastness				Perspiration Fastness							
								Acid				Alkaline			
		Dry	Wet	St.	St. *	St. **	Alt.	St.	St. *	St. **	Alt.	St.	St. *	St. **	Alt.
**5a**	4–5	3–4	3–4	4	4	4	4	4–5	4–5	4–5	4	4–5	4–5	4–5	4
**5b**	4	3–4	3–4	4	4	4	4	4–5	4–5	4–5	4	4–5	4–5	4–5	4
**5c**	4	3–4	3–4	4	4	4	4	4–5	4–5	4–5	4	4–5	4–5	4–5	4
**5d**	4	3–4	3–4	4	4	4	4	4–5	4–5	4–5	4	4–5	4–5	4–5	4
**5e**	4	3–4	3–4	4	4	4	4	4–5	4–5	4–5	4	4–5	4–5	4–5	4

**Table 6:** K/S values of the dyed substrate at 130 °C.

Dye No.	Dyeing Procedure *	K/S	λ_max_	L*	C*	a*	h*	b*
**5a**	Dyebath reuse A	0.50	380	81.05	6.54	−2.86	115.98	5.88
	Dyebath reuse B	0.24	380	81.66	1.45	−1.01	133.81	1.05
**5b**	Dyebath reuse A	0.39	380	81.39	5.48	−2.70	119.57	4.77
	Dyebath reuse B	0.21	380	81.16	0.85	−0.65	139.73	0.55
**5c**	Dyebath reuse A	0.70	380	81.04	11.10	−4.79	115.56	10.01
	Dyebath reuse B	0.21	380	81.45	0.88	−0.78	153.16	0.40
**5d**	Dyebath reuse A	0.71	380	81.91	11.82	−4.76	113.76	10.82
	Dyebath reuse B	0.23	380	81.94	1.71	−0.90	121.87	1.46
**5e**	Dyebath reuse A	0.40	380	81.81	6.07	−2.46	113.94	5.55
	Dyebath reuse B	0.22	380	81.50	0.90	−0.71	142.01	0.56

* Dyebath reuse A: using the dyebath for the first time; dyebath reuse B: using the dyebath for the second time.

**Table 7:** K/S values of the dyed substrate at 100 °C.

Dye No.	Dyeing Procedure	K/S	λ_max_	L*	C*	a*	h*	b*
**5a**	Dyebath reuse A	4.34	380	78.47	39.29	−8.82	102.98	38.28
	Dyebath reuse B	0.46	380	80.26	5.86	−2.86	119.24	5.11
**5b**	Dyebath reuse A	3.83	380	79.25	36.62	−9.92	105.72	35.25
	Dyebath reuse B	0.67	380	80.19	10.31	−4.63	116.65	9.22
**5c**	Dyebath reuse A	1.47	380	80.08	22.42	−8.01	110.94	20.94
	Dyebath reuse B	0.30	380	80.11	2.68	−1.97	137.24	1.82
**5d**	Dyebath reuse A	1.39	380	80.05	22.16	−7.19	108.92	20.96
	Dyebath reuse B	0.47	380	79.65	5.91	−2.36	113.51	5.42
**5e**	Dyebath reuse A	1.20	380	80.06	21.00	−7.67	111.43	19.55
	Dyebath reuse B	0.23	380	79.79	0.90	−0.89	189.73	−0.15

**Table 8:** Fastness properties of the dyed substrate at 130 °C.

Dye No.	Dyeing Procedure	Rubbing Fastness		Washing Fastness				Perspiration Fastness							
								Acid				Alkaline			
		Dry	Wet	St.	St. *	St. **	Alt.	St.	St. *	St. **	Alt.	St.	St. *	St. **	Alt.
**5a**	Dye bath reuse A	4–5	4	4	4	4	4	4–5	4–5	4–5	4	4–5	4–5	4–5	4
	Dye bath reuse B	4	4	4	4	4	4	4–5	4–5	4–5	4	4–5	4–5	4–5	4
**5b**	Dye bath reuse A	3–4	3–4	4	4	4	4	4–5	4–5	4–5	4	4–5	4–5	4–5	4
	Dye bath reuse B	3–4	4	4	4	4	4	4–5	4–5	4–5	4	4–5	4–5	4–5	4
**5c**	Dye bath reuse A	4	4	4	4	4	4	4–5	4–5	4–5	4	4–5	4–5	4–5	4
	Dye bath reuse B	3–4	4	4	4	4	4	4–5	4–5	4–5	4	4–5	4–5	4–5	4
**5d**	Dye bath reuse A	4	4	4	4	4	4	4–5	4–5	4–5	4	4–5	4–5	4–5	4
	Dye bath reuse B	4	4	4	4	4	4	4–5	4–5	4–5	4	4–5	4–5	4–5	4
**5e**	Dye bath reuse A	4–5	4	4	4	4	4	4–5	4–5	4–5	4	4–5	4–5	4–5	4
	Dye bath reuse B	3–4	4	4	4	4	4	4–5	4–5	4–5	4	4–5	4–5	4–5	4

**Table 9:** Fastness properties of the dyed substrate at 100 °C.

Dye No	Dyeing Procedure	Rubbing Fastness		Washing Fastness				Perspiration Fastness							
								Acid				Alkaline			
		Dry	Wet	St.	St. *	St. **	Alt.	St.	St. *	St. **	Alt.	St.	St. *	St. **	Alt.
**5a**	Dye bath reuse A	4	4	4	4	4	4	4–5	4–5	4–5	4	4–5	4–5	4–5	4
	Dye bath reuse B	4–5	4	4	4	4	4	4–5	4–5	4–5	4	4–5	4–5	4–5	4
**5b**	Dye bath reuse A	3–4	4	4	4	4	4	4–5	4–5	4–5	4	4–5	4–5	4–5	4
	Dye bath reuse B	4	4	4	4	4	4	4–5	4–5	4–5	4	4–5	4–5	4–5	4
**5c**	Dye bath reuse A	4	4	4	4	4	4	4–5	4–5	4–5	4	4–5	4–5	4–5	4
	Dye bath reuse B	4–5	4	4	4	4	4	4–5	4–5	4–5	4	4–5	4–5	4–5	4
**5d**	Dye bath reuse A	4	4	4	4	4	4	4–5	4–5	4–5	4	4–5	4–5	4–5	4
	Dye bath reuse B	4–5	4	4	4	4	4	4–5	4–5	4–5	4	4–5	4–5	4–5	4
**5e**	Dye bath reuse A	4	4	4	4	4	4	4–5	4–5	4–5	4	4–5	4–5	4–5	4
	Dye bath reuse B	4–5	4	4	4	4	4	4–5	4–5	4–5	4	4–5	4–5	4–5	4

**Table 10:** The antimicrobial activity of dyes **5a**–**e** against the different test microbes.

Dye No.	Inhibition Zone (mm)			
	*Staphylococcus aureus*	*Escherichia coli*	*Candida albicans*	*Aspergillus niger*
**5a**	24	29	27	0
**5b**	25	22	28	0
**5c**	15	24	19	0
**5d**	28	27	27	0
**5e**	21	25	26	0
Neomycin	34	32	37	0
Cyclohexamide	0	0	0	35

**Table 11:** Antitumor activities of the dyes **5a**–**e**.

Dye No.	Cytotoxic Activity (IC_50_ µg/mL)			
	HepG-2	A-549	HCT-116	MCF-7
**5a**	1.48 ± 0.08	2.91 ± 0.23	3.48 ± 0.14	1.99 ± 0.11
**5b**	99.07 ± 2.88	50.60 ± 2.34	60.39 ± 1.29	27.10 ± 1.82
**5c**	2.48 ± 0.09	5.03 ± 0.67	3.89 ± 0.18	3.39 ± 0.25
**5d**	6.38 ± 0.54	13.67 ± 0.93	8.92 ± 0.87	10.03 ± 1.09
**5e**	9.23 ± 0.78	24.60 ± 1.73	14.28 ± 0.98	21.76 ± 1.43
Cisplatin	3.69 ± 0.27	7.49 ± 0.65	2.54 ± 0.23	5.68 ± 0.44

**Table 12:** In vitro cytotoxic activity against normal cell lines (WI-38) and selectivity index (the ratio between the IC_50_ value of normal cells and the IC_50_ value of cancer cells) for the newly synthesized dyes.

Dye No.	(*CC*_50_ µg/mL) Lung WI-38	Selectivity Index			
		HepG-2	A-549	HCT-116	MCF-7
**5a**	21.23 ± 2.79	14.3	7.3	6.1	10.7
**5b**	241.94 ± 11.34	2.4	4.8	4.0	8.9
**5c**	55.60 ± 4.13	22.4	11.1	14.3	16.4
**5d**	59.82 ± 4.65	9.4	4.4	6.7	6
**5e**	56.91 ± 4.32	6.2	2.3	4	2.6

**Table 13:** Antioxidant activities of dyes **5a–e**.

Dye No.	Antioxidant Activity (IC_50_ µg/mL)
**5a**	40.99 ± 3.12
**5b**	51.87 ± 3.29
**5c**	195.75 ± 4.97
**5d**	15.33 ± 0.61
**5e**	60.18 ± 3.46
Ascorbic acid	10.62 ± 0.84

## Data Availability

All datasets used and analyzed supporting the conclusions of this article are available upon request; further inquiries can be directed to the corresponding authors.
